# Supplementary material for: Predicting natural language descriptions of mono-molecular odorants
Source: Nat Commun. 2018 Nov 26;9:4979. doi: 10.1038/s41467-018-07439-9 (PMC6255800; doi:10.1038/s41467-018-07439-9)
Supplement: Supplementary file 1 — Supplementary Information [file 41467_2018_7439_MOESM1_ESM.pdf]

Supplementary Information

Gutiérrez et al.

Predicting natural language descriptions of mono-molecular odorants

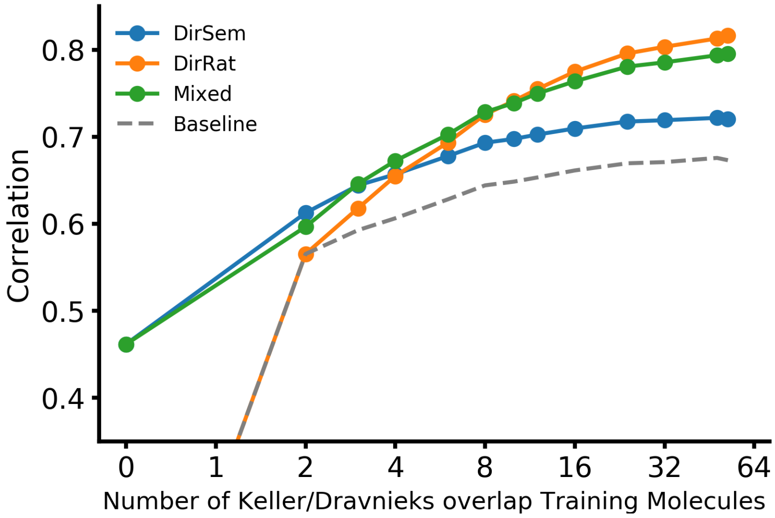

Supplementary Figure 1. Maximum correlation for different models predicting Dravnieks descriptors across 58 overlapping molecules. The performance of the direct semantic (*DirSem* blue dots) and the direct ratings (*DirRat* orange dots) models as well as a the averaged mixed model (green dots), as the number of molecules used in training is increased.

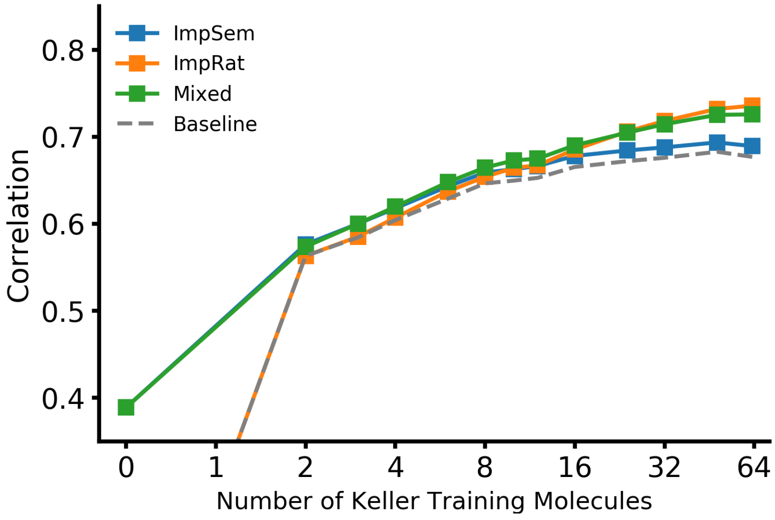

Supplementary Figure 2. Maximum correlation for different models predicting Dravnieks descriptors across non-overlapping molecules. The performance of the imputed semantic (*ImpSem* blue squares) and the imputed ratings (*ImpRat* orange squares) models as well as a the averaged model (green squares), as the number of molecules used in training is increased.

BAKERY	MALTY	0.870324290327
SWEET	SWEET	0.799501672662
FRUIT	PEACH	0.809634562228
FISH	SAUERKRAUT	0.841566522847
GARLIC	SAUERKRAUT	0.807089110708
SPICES	CLOVE	0.78750270191
COLD	COOLING	0.640995565798
SOUR	RANCID	0.85291315729
BURNT	SMOKY	0.611806648709
ACID	CHEMICAL	0.558275863815
WARM	BAKERY	0.629423235915
MUSKY	SWEATY	0.741410635391
SWEATY	RANCID	0.813893445052
AMMONIA	URINE	0.516659287626
DECAYED	PUTRID	0.796734051932
WOOD	PEPPERS	0.439374864874
GRASS	HERBAL	0.569829062717
FLOWER	FLORAL	0.779178189326
CHEMICAL	CARBOLIC	0.65387018244

Supplementary Table1. Maximum correlation between DREAM (left) and Dravnieks (right) descriptors across 58 overlapping molecules.

Supplementary Data 1. Predictions for leave-one-out models in Figure 3 & 4. Including the *DirSem*, *DirMix* and *DirRat* models in first three sheets as well as the predictions from the model developed in the DREAM challenge using molecular descriptors aka *DREAM* in the fourth sheet, and used as input for the *ImpSem*, *ImpMix* and *ImpRat* models in last three sheets.

Supplementary Data 2. Predictions for Paradigm Odors in Figure 5. First sheet are the 83 perceptual descriptors used to describe the 35 paradigm odors and indicated with a *1* when used , *empty spaces* otherwise. Second sheet are the DREAM model predictions for the 35 paradigm odors using their molecular descriptors. Third sheet are for each of the 35 paradigm odors, the ordered 80 perceptual descriptors following the values of the predictions of the semantic model using the DREAM model predictions as input.
