# Supplementary material for: Predicting natural language descriptions of mono-molecular odorants
Source: Nat Commun. 2018 Nov 26;9:4979. doi: 10.1038/s41467-018-07439-9 (PMC6255800; doi:10.1038/s41467-018-07439-9)
Supplement: Supplementary file 5 — Description of Additional Supplementary Files [file 41467_2018_7439_MOESM5_ESM.pdf]

Description of Additional Supplementary Files

Supplementary Data 1. Predictions for leave-one-out models in Figure 3 & 4. Including the DirSem, DirMix and DirRat models in first three sheets as well as the predictions from the model developed in the DREAM challenge using molecular descriptors aka DREAM in the fourth sheet, and used as input for the ImpSem, ImpMix and ImpRat models in last three sheets.

Supplementary Data 2. Predictions for Paradigm Odors in Figure 5. First sheet are the 83 perceptual descriptors used to describe the 35 paradigm odors and indicated with a 1 when used , empty spaces otherwise. Second sheet are the DREAM model predictions for the 35 paradigm odors using their molecular descriptors. Third sheet are for each of the 35 paradigm odors, the ordered 80 perceptual descriptors following the values of the predictions of the semantic model using the DREAM model predictions as input.
